# Incidence and Risk Factors for Cerebrovascular-Specific Mortality in Patients with Colorectal Cancer: A Registry-Based Cohort Study Involving 563,298 Patients

**DOI:** 10.3390/cancers14092053

**Published:** 2022-04-19

**Authors:** Zhi-Hui Dai, Ming Tang, Yun-Liang Chen, Tao-Lan Zhang, Jing Li, Guo-Hua Lv, Yi-Guo Yan, Zhi-Hua Ouyang, Wei Huang, Ming-Xiang Zou

**Affiliations:** 1 Department of Orthopedics, Wenzhou Medical University Affiliated Dongyang Hospital, Dongyang 330783, China; airdaizhihui@gmail.com; 2 Department of Spine Surgery, The First Affiliated Hospital, Hengyang Medical School, University of South China, Hengyang 421001, China; tmlhr0122@163.com (M.T.); yanyiguo@live.cn (Y.-G.Y.); yzh175@163.com (Z.-H.O.); 3 Shenzhen Audaque Data Technology Company, Shenzhen 440300, China; yunliang_chen@audaque.com; 4 Department of Scientific Research and Discipline Development, The First Affiliated Hospital, Hengyang Medical School, University of South China, Hengyang 421001, China; csu_ztl@163.com; 5 Department of Spine Surgery, The Second Xiangya Hospital, Central South University, Changsha 410011, China; jingli1969@csu.edu.cn (J.L.); xylgh1031@csu.edu.cn (G.-H.L.); 6 Health Management Center, The First Affiliated Hospital, Hengyang Medical School, University of South China, Hengyang 421001, China

**Keywords:** colorectal cancer, cerebrovascular-specific mortality, cerebrovascular-specific diseases, incidence, risk factors

## Abstract

**Simple Summary:**

Previous studies have shown that the occurrence of cerebrovascular-specific diseases was common in cancer patients. However, the association between colorectal cancer and cerebrovascular-specific diseases remains to be fully elucidated. In this large-population cohort study, we found that the mortality of cerebrovascular-specific diseases mortality in colorectal cancer patients was significantly higher than the general US population. In addition, we investigated several potential predictors of cerebrovascular-specific diseases mortality in colorectal cancer. This study may be useful for the prevention, risk stratification and therapeutic optimization of cerebrovascular-specific diseases in colorectal cancer patients.

**Abstract:**

Background: Colorectal cancer (CRC) is one of the most prevalent diseases and the second leading cause of death worldwide. However, the relationship between CRC and cerebrovascular-specific mortality (CVSM) remains elusive, and less is known about the influencing factors associated with CVSM in CRC. Here, we aimed to analyze the incidence as well as the risk factors of CVSM in CRC. Methods: Patients with a primary CRC diagnosed between 1973 and 2015 were identified from the Surveillance Epidemiology and End Results database, with follow-up data available until 31 December 2016. Conditional standardized mortality ratios were calculated to compare the incidence of CVSM between CRC patients and the general U.S. population. Univariate and multivariate survival analyses with a competing risk model were used to interrogate the risk factors for CVSM. Results: A total of 563,298 CRC individuals were included. The CVSM in CRC patients was significantly higher than the general population in all age subgroups. Among the competing causes of death in patients, the cumulative mortality caused by cerebrovascular-specific diseases steadily increased during the study period. While age, surgery, other/unknown race and tumors located at the transverse colon positively influenced CVSM on both univariate and multivariate analyses, male patients and those who had radiotherapy, chemotherapy, a more recent year (2001–2015) of diagnosis, a grade II or III CRC, rectal cancer, or multiple primary or distant tumors experienced a lower risk of CVSM. Interpretation: Our data suggest a potential role for CRC in the incidence of CVSM and also identify several significant predictors of CVSM that may be helpful for risk stratification and the therapeutic optimization of cerebrovascular-specific diseases in CRC patients.

## 1. Introduction

Colorectal cancer (CRC) ranks as the third most common malignant cancer and the second leading cause of cancer deaths worldwide, with 1.9 million newly diagnosed CRC cases and 935,000 deaths reported in 2020 [1,2]. Due to the growth and aging of the population, the number of patients who die from CRC continues to increase; this poses a great medical and economic burden for the nation [3]. Therefore, it is imperative to further optimize therapeutic decisions and risk stratification to reduce the mortality of patients.

Previous studies have shown that the occurrence of cerebrovascular-specific diseases (CVSDs) is common in cancer patients [4]. For instance, it has been demonstrated that the incidence rates of stroke in patients with lung cancer and breast cancer were 5.1% and 1.5%, respectively [5]. A similar study indicated a cumulative incidence of 7% for ischemic stroke in Hodgkin lymphoma [6]. Despite these findings, the current evidence regarding the association between CRC and CVSDs is limited. Moreover, there is currently a lack of studies (especially those with large sample size and long-term follow-up data) comprehensively dealing with factors related to the rate of cerebrovascular-specific mortality (CVSM) in CRC.

Researchers have verified that CRC is a heterogeneous pathology characterized by a gut microbiota disorder [7,8]. Furthermore, recent studies have suggested that aberrant gut microbiota components can lead to vascular atherosclerosis, thereby promoting the incidence of cardiovascular-related events [9,10]. However, whether the dysregulated gut microbiota in CRC could also contribute to an increased risk of developing CVSDs deserves further exploration. Previously published reports have indicated that CVSDs constitute a major factor influencing the mortality of CRC patients [11,12]. Given the high incidence and mortality rate of CRC in the general population, it is important to identify CRC survivors who are at an elevated risk of CVSM to stratify patients and guide therapeutic optimization, thereby improving the survival and quality of life of patients. In this study, we aimed to analyze the incidence and risk factors for CVSM in a large CRC patient cohort. To this end, we first collected CRC patients from the Surveillance, Epidemiology, and End Results (SEER) database and compared their CVSM to that of the general population. We then used univariate and multivariate analyses with a competing risk model to identify the predictive factors of CVSM for these patients.

## 2. Methods and Materials

### 2.1. Data Sources

In this registry-based retrospective cohort study, we analyzed colorectal cancer (CRC) patient data from the SEER database [13]. This database consists of 21 regional cancer registries, which cover 37% of the U.S. population. It provides comprehensive demographic and detailed cancer-related information for a large population of patients. In addition, the SEER database standardizes clinical features (such as diagnosis, treatment, causes of death, tumor grade, tumor stage and tumor site) codes for patients by International Classification Diseases (ICD) every year. To ensure comparability, we converted all ICD codes to the latest ICD-10 using the SEER database conversion function. In addition, the reference cohort represented by the general U.S. population was obtained from the Centers for Disease Control and Prevention (CDC) database [14].

### 2.2. Study Population

**Figure 1:**
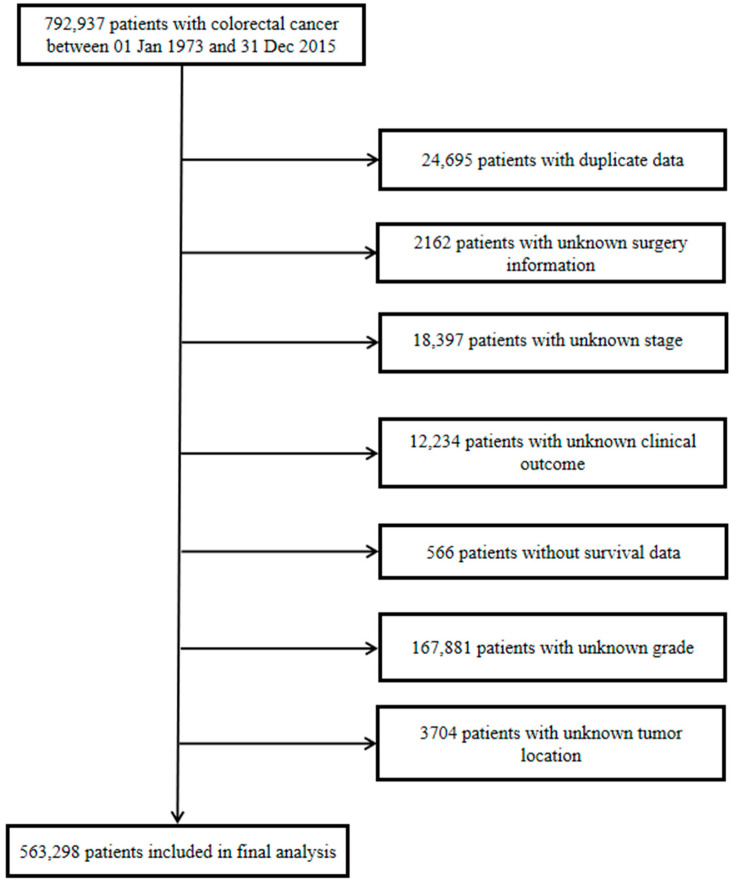
Flowchart of the selection of study subjects.

CRC patients were identified as those who were newly diagnosed with colon or rectal cancer between 1 January 1973 and 31 December 2015 in the SEER database. According to the ICD-10 codes, colon or rectal cancer included the following: cecum (C18.0), appendix (18.1), ascending colon (C18.2), hepatic flexure of colon (C18.3), transverse colon (C18.4), splenic flexure of colon (C18.5), descending colon (C18.6), sigmoid colon (C18.7), overlapping lesion of colon (C18.8), rectosigmoid junction (C19.9) and rectum (C20.9). Patients with an unknown tumor location (C18.9) were excluded from the study. We screened a subset of CRC patients by exclusion criteria, and all procedures are shown in Figure 1.

We retrieved the clinical data of each patient from the database, which included the following: year of CRC diagnosis (≤2000/2001–2005/2006–2015), age at diagnosis (<50/50–64/65–75/≥75 years), sex (female/male), race (white/black/unknown), tumor grade (I–IV), tumor stage (in situ/localized/regional/distant), number of primary tumors (one/multiple), radiotherapy, chemotherapy and tumor site. For the purpose of analysis with a large enough sample size, data on the year of diagnosis and patient age were separated into different subgroups as suggested previously [15]. ‘Multiple tumors’ was defined by whether a patient was diagnosed with more than one primary tumor when alive, irrespective of whether the disease was CRC or not. Tumor grade and stage were defined by the American Joint Committee on Cancer (AJCC) 6th edition from the SEER database. Tumor sites were classified according to colorectal anatomy [16]. CVSM was defined as the time from the diagnosis of CRC to patient death caused by CVSDs, which included hemorrhage (I610–629), thrombosis or infarction (I630–639), occlusion of cerebral artery (I652 and I669), transient ischemic attack (G459), cerebrovascular insult (I649 and I679) and late effects of cerebrovascular insult (I692) according to the ICD-10 codes from the database. Patients were followed-up until death or the end of observation in December 2016, whichever occurred first.

### 2.3. Statistical Analysis

Continuous variables are presented as the mean ± standard deviation and were analyzed by the Student’s *t*-test. Categorical variables are expressed as numbers and percentages (%) and were analyzed using the chi-square test or Wilcoxon’s rank sum test, when appropriate. We estimated the cumulative mortality for all causes among CRC patients after diagnosis with a competing risk model with mortalities from CVSDs, CRC, other cancers and other noncancer diseases as competing risks. Specifically, the crude cumulative mortality functions were computed and plotted for cause-specific deaths overall and stratified by clinical factors (univariate analysis) to display the probability of experiencing an endpoint of interest in the presence of competing events, and differences in cumulative mortality among subgroups were analyzed by Gary’s test. This method was adopted due to its widespread use in analyzing data with competing events [17,18,19] and its ability to overcome the overestimation of the absolute risk of the event of interest when compared to the standard Kaplan–Meier method. This competing risk model was also used to identify independent influencing factors associated with CVSM in CRC patients, in which multivariate survival analyses (only including significant factors in the univariate analysis) were performed by calculating the cumulative mortality and hazard ratios (HRs) between different subgroups for each predictor. Spearman’s correlation test was applied to evaluate the potential relationship between variables included in multivariate analyses. The absolute value for a Spearman correlation coefficient of 0.7–1 was regarded as a strong association, 0.5–0.7 as a moderate association, 0.3–0.5 as a weak association and 0–0.3 as no association [20,21].

We calculated the CVSDs and overall conditional standardized mortality ratio (cSMR) in CRC patients relative to the general U.S. population stratified by patient age (including 8 subgroups: < 50, 50–54, 55–59, 60–64, 65–69, 70–74, 75–79 and 80–84 years) and year of diagnosis (including 3 subgroups: ≤2000, 2001–2005 and 2006–2015). The cSMR was defined as the ratio of CVSD-specific or overall deaths in the CRC group to the estimated number of deaths in the general U.S. population. Patients aged more than 84 years were not included for analysis due to the lack of these data in the general population from the CDC database. We determined the age classifications for two reasons. First, given the small number of younger CRC patients, we divided them into a single group to allow for statistical effectiveness. Second, the official mortality statistics for the U.S. population were categorized in exactly the same 5-year steps, making it easier for us to conduct comparative analyses.

All analyses were conducted using R version 3.5.1 (R Foundation for Statistical Computing, Vienna, Austria). The 95% confidence interval (95% CI) and *p* value were estimated as previously described [22,23]. All tests were two-sided, and a *p* value of less than 0.05 was considered statistically significant.

## 3. Results

### 3.1. Patient Characteristics

A total of 563,298 CRC patients were included in this study, with a median age of 68.6 ± 13.1 years. The median follow-up time was 6.67 ± 6.48 years. The sex ratio was basically equal (male, 50.9%). The majority of patients were white (83.3%) and diagnosed after the age of 75 years (36.7%). Most patients had one primary tumor (70.7%), a regional tumor stage (41.7%) and a tumor grade of II (66.7%). A total of 94.1% of the patients received surgery, while a small proportion of the patients had chemotherapy (11.6%) or radiotherapy (29.1%). All patient characteristics are detailed in Table 1. In addition, our data indicated a statistically significant difference in clinical features between CVSM and non-CVSM subgroups of CRC patients (Supplementary Table S1).

### 3.2. Cumulative Mortality

**Figure 2:**
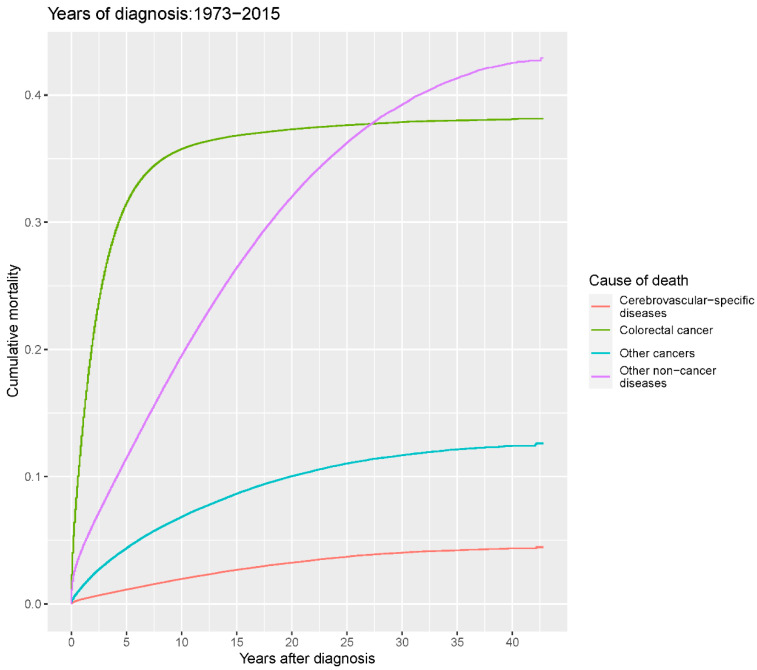
Cumulative cause-specific mortality among colorectal cancer patients.

**Figure 3:**
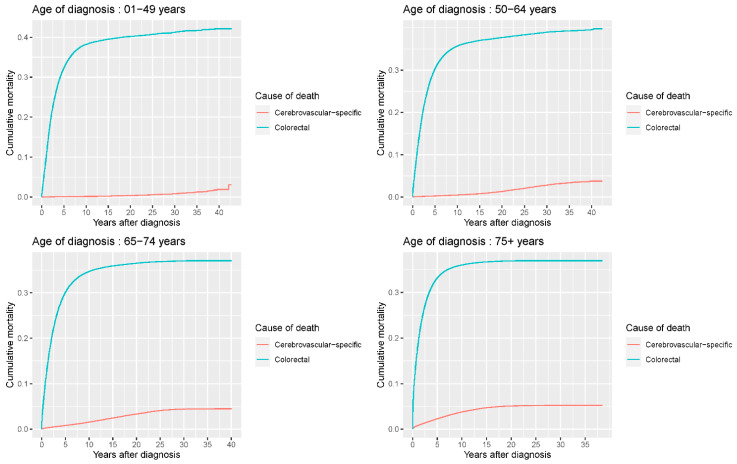
Univariate analyses using a competing risk model showed a significant association of patient age with CVSM in CRC patients (*p* < 0.0001). Patients were separated into four subgroups (<50/50–64/65–75/≥75 years) according to age, and crude cumulative mortality functions were computed and plotted for cerebrovascular-specific and colorectal cancer-specific mortality for each subgroup to display the probability of experiencing CVSM- or CRC-specific deaths in the presence of competing events. Differences in cumulative mortality among different subgroups were analyzed by Gary’s test.

**Figure 4:**
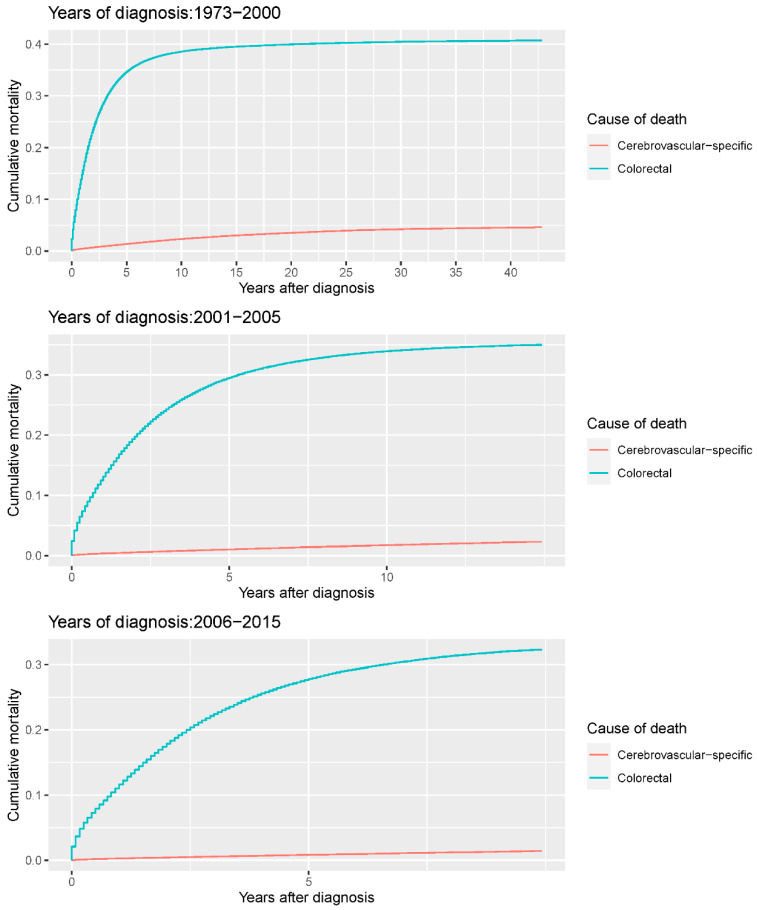
Cumulative cerebrovascular-specific and colorectal cancer-specific mortality stratified by year at diagnosis. Univariate analyses using a competing risk model were performed to test the relationship between year of diagnosis and CVSM in CRC patients (*p* < 0.0001), and differences in cumulative mortality among different subgroups were analyzed by Gary’s test.

**Figure 5:**
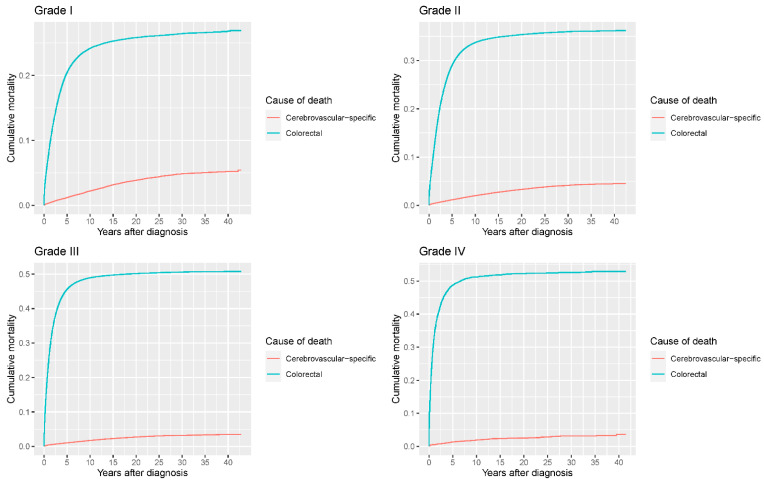
Cumulative cerebrovascular-specific and colorectal cancer-specific mortality stratified by tumor grade. Univariate analyses using a competing risk model were performed to test the relationship between tumor grade and CVSM in CRC patients (*p* < 0.0001), and differences in cumulative mortality among different subgroups were analyzed by Gary’s test.

**Figure 6:**
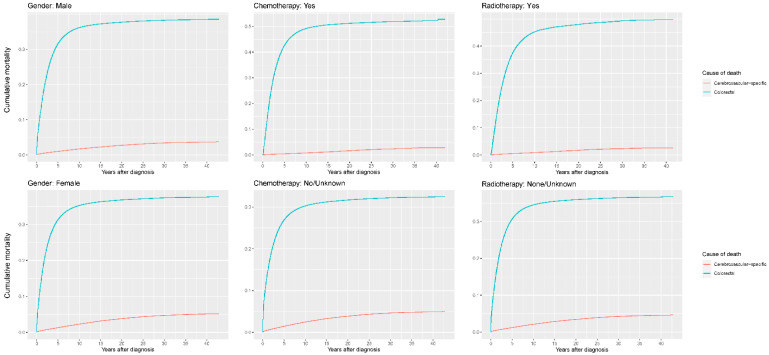
Cumulative cerebrovascular-specific and colorectal cancer-specific mortality stratified by gender, chemotherapy and radiotherapy. Univariate analyses using a competing risk model were performed to test the relationship between the clinical factors and CVSM in CRC patients (*p* < 0.0001), and differences in cumulative mortality among different subgroups were analyzed by Gary’s test.

**Figure 7:**
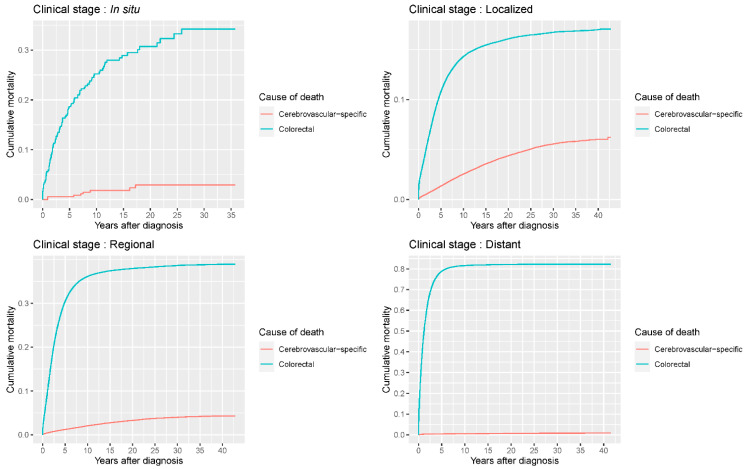
Cumulative cerebrovascular-specific and colorectal cancer-specific mortality stratified by tumor stage. Univariate analyses using a competing risk model were performed to assess the relationship between tumor clinical stage and CVSM in CRC patients (*p* < 0.0001), and differences in cumulative mortality among different subgroups were analyzed by Gary’s test.

**Figure 8:**
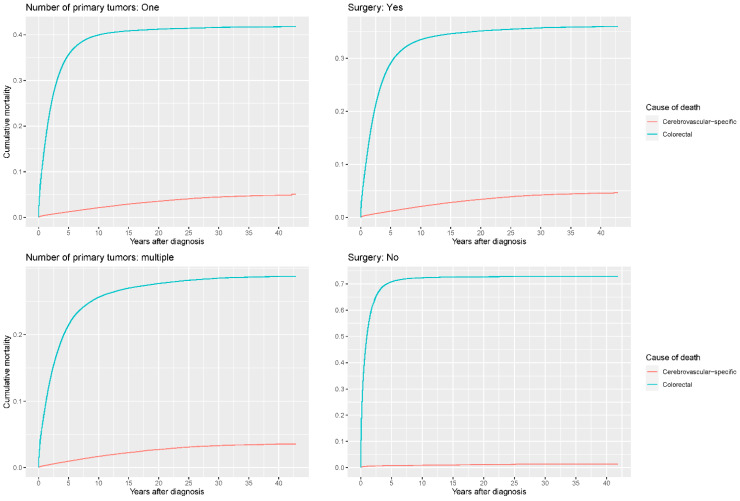
Cumulative cerebrovascular-specific and colorectal cancer-specific mortality stratified by number of primary tumors and surgery. Univariate analyses using a competing risk model were performed to test the relationship between these factors and CVSM in CRC patients (*p* < 0.0001), and differences in cumulative mortality among different subgroups were analyzed by Gary’s test.

**Figure 9:**
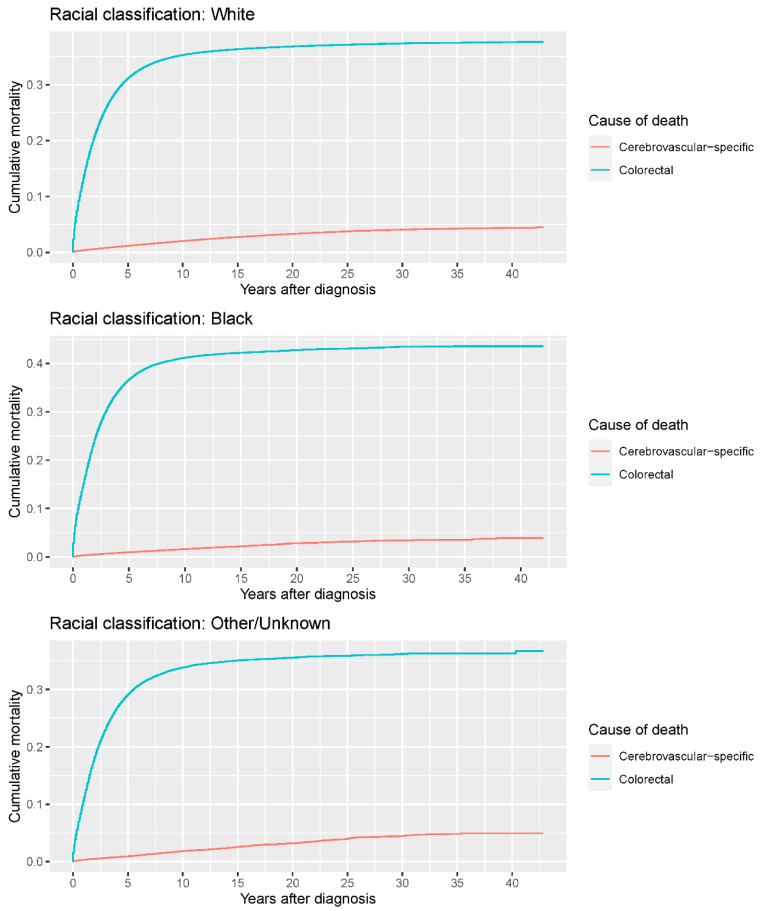
Cumulative cerebrovascular-specific and colorectal cancer-specific mortality stratified by race. Univariate analyses using a competing risk model were performed to test the relationship between race and CVSM in CRC patients (*p* < 0.0001), and differences in cumulative mortality among different subgroups were analyzed by Gary’s test.

**Figure 10:**
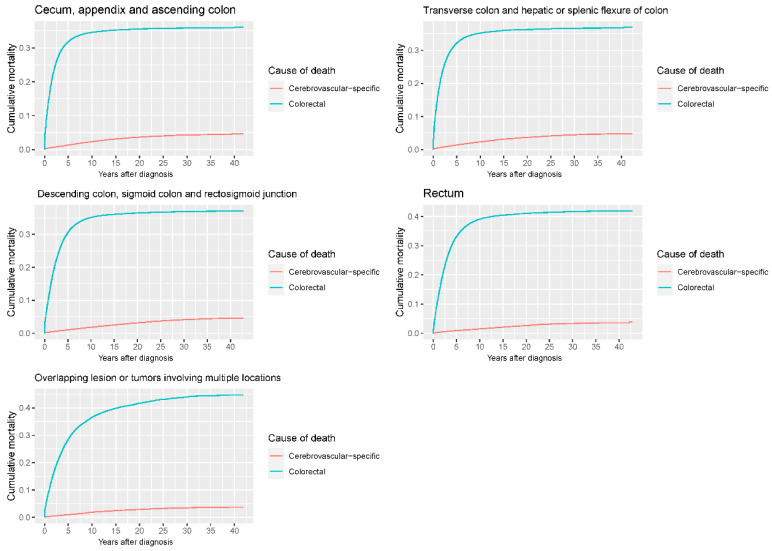
Cumulative cerebrovascular-specific and colorectal cancer-specific mortality stratified by tumor site. Univariate analyses using a competing risk model were performed to test the relationship between tumor location and CVSM in CRC patients (*p* < 0.0001), and differences in cumulative mortality among different subgroups were analyzed by Gary’s test.

Our study found that cumulative mortality from CVSDs slightly increased from 1973 to 2015. CRC had the highest cumulative mortality, followed by other noncancer causes of death in the early period of 1973–2015. However, this trend went in the opposite direction in the middle and late periods (Figure 2). In the univariate analyses, our data showed that cumulative mortality from CVSDs was positively associated with age (Figure 3) and the year of CRC diagnosis (Figure 4), while the tumor grade had an inverse correlation with CVSM (Figure 5). In addition, male patients and those who received chemotherapy or radiotherapy experienced a lower cumulative mortality from CVSDs than their counterparts (Figure 6). Similar results were observed in patients with distant CRC (Figure 7) or multiple primary tumors, as well as in those who were not treated with surgery (Figure 8). The relationships between CVSM and race or tumor site are shown in Figure 9 and Figure 10, respectively.

### 3.3. Conditional Standardized Mortality Ratio

The comparative results of cSMR data between CRC patients and the general population are displayed in Table 2. Our study revealed that the CVSM and overall mortality in CRC patients were significantly higher than those in the general U.S. population in all age and year of diagnosis subgroups. In the analysis of cSMR for CVSDs, our research discovered that younger CRC patients (age < 50 years) harbored the highest cSMR, which was 13 times higher than that of elderly patients aged 80–84 years (83.14, 95% CI: 69.99–97.42 vs. 6.91, 95% CI: 6.66–7.17). In contrast, an inverse association was observed for patients diagnosed in the most recent years and the cSMR of CVSDs (Supplementary Table S2). In fact, as age is known to have a positive association with CVSD-specific deaths, it is easy to understand that the cSMR was likely low in elderly patients, whereas this value could be high for younger patients because the estimated number of deaths in the normal population is relatively small for this age subgroup.

### 3.4. Cause-Specific Hazard Ratios (HRs)

The connection between individual prognostic factors and CVSM among CRC patients is displayed in Table 3. Correlation analyses revealed no or weak interactions among the factors included in the multivariate model (Supplementary Table S3), suggesting the appropriateness of our model. Multivariate analyses using a competing risk model showed that CVSM was positively correlated with age. The HRs were 3.098 (95% CI: 2.617–3.667), 6.666 (95% CI: 5.654–7.859) and 10.951 (95% CI: 9.299–12.897) for patients aged 55–64 years, 65–74 years and ≥75 years old, respectively. However, the HRs for the patients who received radiotherapy or chemotherapy were 0.849 (95% CI: 0.783–0.921) and 0.653 (95% CI: 0.616–0.691), respectively, suggesting a protective effect for these factors against CVSM. Similar results were also observed in patients with multiple primary tumors (0.685, 95% CI: 0.659–0.712) and distant CRC (0.298, 95% CI: 0.149–0.598). By contrast, patients undergoing surgery faced a 1.558-times higher risk of CVSM than those who were not surgically treated. In addition, our analyses revealed that patients having grade II or III tumors and those with rectal tumors experienced a reduced risk of CVSM compared to patients with grade I tumors and harboring disease located at the cecum, appendix and ascending colon. However, patients of other or unknown race and those with lesions involving the transverse colon and hepatic or splenic flexure of colon displayed an elevated risk of mortality due to CVSDs compared to their counterparts.

## 4. Discussion

In this study, we analyzed the CVSM in a large registry-based CRC cohort and compared this outcome with that in the general population. We also characterized the prognostic factors of CVSM in CRC patients. We found that the CVSM in CRC patients was significantly higher than that in the general population. Moreover, we identified several important clinical factors affecting CVSM in the patients. These data may provide a comprehensive understanding of CVSM in CRC and help guide risk stratification and therapeutic optimization for CVSDs in this population.

To date, this is the largest individual patient-level study to explore long-term CVSM among CRC patients. Our study revealed that the CVSM in CRC patients was increased compared to that in the general population, which was in line with previous studies claiming that malignant tumor lesions were associated with the development of CVSDs. [5,12,24] It is well known that the brain–gut axis is a bidirectional link between the brain and the gastrointestinal tract [25]. Published data have indicated that the gut microbiota, as an important component of the brain–gut axis, can contribute to CVSDs by mediating many pathways, including vascular inflammation, lipopolysaccharide signaling and endothelial and immune cell functions [26,27,28,29]. In addition, studies have demonstrated that abnormal vitamin K and trimethylamine N-oxide levels produced by gut microbiota can lead to several pathologies, including thrombosis, atherosclerosis and coagulopathy, [30,31,32,33] all of which are linked with an increased risk of CVSDs occurrence. Given that the gut microbiota is aberrant in CRC, [34,35,36] these data suggest that the gut microbiota may play a pivotal role in CVSD development among CRC patients. Future animal studies involving various gut microbiota models may be helpful in further clarifying this correlation and the potential mechanisms between them.

Moreover, our study also found that age displayed a positive association with CVSDs in CRC, in agreement with previous studies [15]. This condition may likely be caused by the fact that older patients usually have worse elasticity of blood vessel walls than younger patients. In addition, elderly patients suffering from other comorbidities (such as hypertension and diabetes) may be prone to cerebrovascular accidents [37]. Notably, our analysis revealed that young CRC patients (<50 years old) experienced the highest risk of CVSM compared to the general population, which may be caused by unhealthy dietary lifestyles and a lack of physical activity in these survivors [38]. Additionally, our data indicated that the year of diagnosis was negatively correlated with CVSM. This result may be attributed to the improved ability to diagnose and treat CVSDs among CRC patients in recent years [39]. Furthermore, our study also demonstrated that patients of other/unknown race had a slightly higher risk of CVSM than white patients. However, this outcome may be biased by the relatively small number of patients in the other/unknown race subgroup and requires further clarification as no complete race information was available for this patient cohort.

Interestingly, our data hinted that female CRC patients had a higher CVSM, which was contradictory to previous reports proving that estrogens provide a protective effect against vascular disease [40,41]. This inconsistency may be because most female CRC patients are diagnosed at an older age when their ovarian function is remarkably compromised, thus decreasing estrogen production. Another major finding was that both chemotherapy and radiotherapy favorably influenced the CVSM of CRC patients. Preceding observations have confirmed that the intensity of the immune response (specifically including the level of IFN-γ, as well as CD4^+^ and CD8^+^ cell densities) is positively associated with the development of CVSDs [42,43]. Considering this, we speculate that CRC-associated treatments may affect CVSD outcomes by regulating the inflammatory response, thus leading to a reduction in proinflammatory factors entering the blood–brain barrier. Moreover, our study revealed that CRC patients who underwent surgery had an increased risk of CVSM, in accordance with preceding data indicating that hemodynamic changes during the perioperative period may cause the thrombus to fall off, thereby leading to stroke in CRC patients [44,45]. Another possible explanation for such an association is that surgical procedures, especially in patients with advanced CRC, may trigger tumor cells to release more pro-inflammatory factors entering the blood–brain barrier and therefore promote the occurrence of CVSDs. Considering this finding, we recommend that with intraoperative hemodynamic monitoring, surgeons should also reduce operation time, surgical trauma and the amount of bleeding to decrease the risk of CVSD in CRC patients. In addition, for patients with a high risk of CVSD (such as those who are older or have atrial fibrillation or venous thrombosis), prophylactic medication may be administered in a timely fashion after consultation with physicians. Finally, when suspicious of CVSD occurrence, comprehensive examinations (such as cranial magnetic resonance imaging and/or magnetic resonance angiography, as well as systematic inflammation level evaluation) should be performed and then appropriate treatment should be arranged if necessary.

Furthermore, our results showed that patients with multiple tumors had a lower CVSM than their counterparts. This phenomenon is easy to understand, as patients in this subgroup are more likely to receive relevant interventions for CVSDs, thereby resulting in a decreased CVSM rate. It has been described that circulating tumor cells and their extracellular vesicles may lead to CVSDs in patients with metastatic diseases by disrupting the blood–brain barrier and inducing inflammation-associated injuries of vascular endothelial cells [46,47]. Inconsistent with this outcome, however, we uncovered that CRC patients harboring distant or metastatic entities experienced a reduced risk of CVSM. Patients with advanced CRC may die before the incidence of CVSDs because of the short survival time. This finding may also provide an explanatory basis for the favorable CVSM among patients with grade II or III tumors, as advanced pathology grade is reported to be associated with malignant tumor behavior (such as promoting tumor budding, angiogenesis and epithelial-mesenchymal transition) and thus dismal patient survival [48,49]. Apart from this, it should be noted that the small number of patients assigned to the in situ CRC subtype may lower the statistical power and thus introduce bias to our outcome. Taken together, these data may provide an explanation for the inverse relationship between patients’ CVSM and distant CRC. Another major finding was that patients with rectal cancer possessed a lower CVSM, while those who had a tumor in the transverse colon and hepatic or splenic flexure of colon suffered from an increased risk of CVSD mortality. This phenomenon may emerge because tumors located at the transverse colon are prone to infiltrate vascular structures and then cause their dysfunction (by releasing CRC-associated inflammatory factors or embolus), thereby leading to a high probability of CVSM occurrence.

### Strengths and Limitations

The strengths of this study are the large sample size, the stratified analyses by detailed clinicopathological variables and the long follow-up time. However, there are several limitations in our study. First, due to the lack of information on comorbidities (such as hypertension, diabetes and hyperlipidemia), sociodemographic characteristics (such as occupation and BMI) and lifestyle (drinking, smoking history and exercise) related to CVSD in the SEER database or non-CRC cohort, we were not able to include these factors in subsequent statistical analyses or to perform a comparative analysis between the CRC and the normal cohorts. A similar limitation on data collection in molecular features, type of chemotherapy or radiotherapy (specifically including therapeutic duration and dosage) or resection modality in the SEER database may also compromise the generalizability of our results. In addition, the same data restriction precludes us from performing further subgroup analysis stratified by the specific cause (ischemic and hemorrhagic) of CVSM to produce more accurate data on cerebrovascular-specific events in CRC and the potential relationship between them. Future high-quality studies with complete CVSD subtype data (possibly using other databases such as SEER-Medicare or QResearch) are highly needed to make this subgroup analysis possible and further clarify the effect of CVSD type on CVSM in CRC. Finally, additional studies are required to further unveil the precise mechanism of how CVSM could be increased in CRC patients.

## 5. Conclusions

The present study analyzed the incidence of CVSM in CRC patients and systematically investigated the influencing factors associated with CVSD events. We found that the CVSM of CRC patients was significantly higher than that of the general U.S. population and identified several potential predictors of CVSM in the population. These data may be useful for the prevention, risk stratification and therapeutic optimization of CVSM in CRC patients. Further prospective studies involving large sample sizes with more complete clinical and molecular data are needed to confirm our current findings.

## Figures and Tables

**Table 1:** Basic characteristics of included patients with colorectal cancer at time of diagnosis.

Factors	Number of Patients (%)
*N*	563,298
Age (continuous, mean ± SD)	68.6 ± 13.1
Age (years, groups)	
<50	47,947 (8.5)
50–64	150,201 (26.7)
65–74	158,222 (28.1)
75+	206,928 (36.7)
Year of diagnosis	
≤2000	269,323 (47.8)
2001–2005	148,725 (26.4)
2006–2015	145,250 (25.8)
Sex	
Female	276,592 (49.1)
Male	286,706 (50.9)
Race	
White	469,427 (83.3)
Black	53,364 (9.5)
Other/unknown	40,507 (7.2)
Grading	
I	69,849 (12.4)
II	375,542 (66.7)
III	109,875 (19.5)
Ⅳ	8032 (1.4)
Stage	
In situ	356 (0.1)
Localized	224,781 (39.9)
Regional	234,669 (41.7)
Distant	103,492 (18.4)
Tumor site	
Rectum	99,924 (17.7)
Rectosigmoid junction	50,683 (9.0)
Overlapping lesion or tumor involving multiple locations	26,848 (4.9)
Sigmoid colon	123,198 (21.9)
Descending colon	24,961 (4.4)
Splenic flexure of colon	14,779 (2.6)
Transverse colon	36,841 (6.5)
Hepatic flexure of colon	20,629 (3.7)
Ascending colon	67,767 (12.0)
Appendix	3987 (0.7)
Cecum	93,681 (16.6)
Cause of death	
Alive	155,753 (27.7)
Colorectal cancer	202,872 (36.0)
Cerebrovascular-specific diseases	14,600 (2.6)
Other cancer	46,648 (8.3)
Other non-cancer diseases	143,425 (25.5)
Radiotherapy	
Yes	65,352 (11.6)
No/unknown	497,946 (88.4)
Chemotherapy	
Yes	163,975 (29.1)
No/unknown	399,323 (70.9)
Number of primary tumors	
One	398,107 (70.7)
Multiple	165,191 (29.3)
Surgery	
Yes	530,282 (94.1)
No	33,016 (5.9)
Duration of follow-up (years, mean ± SD)	6.67 ± 6.48

SD, standard deviation.

**Table 2:** Age-specific and overall standardized mortality ratios for the years 1999–2015 among colorectal cancer patients relative to the U.S. standard population.

AgeGroup	cSMR Cerebrovascular-Specific	*p*-Value	cSMR Overall	*p*-Value
<50	83.14 (69.99–97.42)	**<0.0001**	371.66 (366.75–376.62)	**<0.0001**
50–54	21.76 (18.81–24.92)	**<0.0001**	97.64 (96.23–99.06)	**<0.0001**
55–59	26.54 (24.20–29.00)	**<0.0001**	76.96 (76.04–77.88)	**<0.0001**
60–64	24.56 (22.96–26.22)	**<0.0001**	58.68 (58.09–59.27)	**<0.0001**
65–69	20.92 (19.88–21.99)	**<0.0001**	43.20 (42.82–43.57)	**<0.0001**
70–74	16.37 (15.72–17.04)	**<0.0001**	30.66 (30.41–30.90)	**<0.0001**
75–79	10.64 (10.26–11.03)	**<0.0001**	20.80 (20.64–20.96)	**<0.0001**
80–84	6.91 (6.66–7.17)	**<0.0001**	13.58 (13.47–13.69)	**<0.0001**

cSMR, conditional standardized mortality ratio; Bold values indicate *p* < 0.05.

**Table 3:** Cause-specific hazard and 95% confidence intervals for cerebrovascular-related mortality among colorectal cancer patients.

Factors	Number of Patients	Cause-Specific Hazards Ratios	
		Cerebrovascular	*p*-Value
Age at diagnosis (years)			
<50	47,947	1.000 (ref.)	
50–64	150,201	3.098 (2.617–3.667)	**<0.0001**
65–74	158,222	6.666 (5.654–7.859)	**<0.0001**
≥75	206,928	10.951 (9.299–12.897)	**<0.0001**
Sex			
Male	286,706	1.000 (ref.)	
Female	276,592	1.223 (1.183–1.264)	**<0.0001**
Surgery			
No	33,016	1.000(ref.)	
Yes	530,282	1.558 (1.385–1.754)	**<0.0001**
Radiotherapy			
No/unknown	497,946	1.000 (ref.)	
Yes	65,352	0.849 (0.783–0.921)	**<0.0001**
Chemotherapy			
No/unknown	399,323	1.000 (ref.)	
Yes	163,975	0.653 (0.616–0.691)	**<0.0001**
Year of diagnosis			
≤2000	269,323	1.000 (ref.)	
2001–2005	148,725	0.610 (0.586–0.636)	**<0.0001**
2006–2015	145,250	0.451 (0.428–0.475)	**<0.0001**
Number of primary tumors			
One	398,107	1.000 (ref.)	
Multiple	165,191	0.685 (0.659–0.712)	**<0.0001**
Stage			
In situ	356	1.000 (ref.)	
Localized	224,781	1.361 (0.682–2.715)	0.3800
Regional	234,669	1.120 (0.561–2.236)	0.7500
Distant	103,492	0.298 (0.149–0.598)	**0.0006**
Grading			
I	69,849	1.000 (ref.)	
II	375,542	0.953 (0.910–0.998)	**0.0410**
III	109,875	0.892 (0.840–0.946)	**0.0002**
IV	8032	1.023 (0.875–1.196)	0.7800
Race			
White	469,427	1.000 (ref.)	
Black	53,364	1.019 (0.958–1.085)	0.5500
Other/Unknown	40,507	1.110 (1.039–1.186)	**0.0019**
Tumor site			
Cecum, appendix and ascending colon	165,435	1.000 (ref.)	
Transverse colon and hepatic or splenic flexure of colon	72,249	1.061 (1.008–1.116)	**0.0240**
Descending colon, sigmoid colon and rectosigmoid junction	198,842	0.968 (0.930–1.008)	0.1200
Rectum	99,924	0.900 (0.851–0.952)	**0.0002**
Overlapping lesion or tumors involving multiple locations	26,848	1.057 (0.972–1.149)	0.1900

Bold values indicate *p* < 0.05.

## Data Availability

All data relevant to the study are included in the article or uploaded as supplementary information.

## Supplementary Materials

The following supporting information can be downloaded at: https://www.mdpi.com/article/10.3390/cancers14092053/s1, Supplementary Table S1: Comparison of patient characteristics in CVSM and non-CVSM subgroups of CRC patients; Supplementary Table S2: Year of diagnosis-specific and overall standardized mortality ratios for the years 1999–2015 among colorectal cancer relative to the USA standard population; Supplementary Table S3: Correction between individual prognostic factors of CVSM among CRC patients.
