# Supplementary material for: Incidence and Risk Factors for Cerebrovascular-Specific Mortality in Patients with Colorectal Cancer: A Registry-Based Cohort Study Involving 563,298 Patients
Source: Cancers (Basel). 2022 Apr 19;14(9):2053. doi: 10.3390/cancers14092053 (PMC9105882; doi:10.3390/cancers14092053)
Supplement: Supplementary file 1 [file cancers-14-02053-s001.zip › cancers-1674451-supplementary.pdf]

Supplementary Table S1. Comparison of patient characteristics in CVSM and non-CVSM subgroups of CRC patients

Factors	CVSM	Non-CVSM	*P*-value
N	14600	548698	<0.0001
Age (continuous, mean ± SD)	76.1 ± 9.60	68.4 ± 13.1	<0.0001
Survival time (months)	91.4 ± 80.6	79.7 ± 77.6	<0.0001
Follow-up time (years)	7.59 ± 6.72	6.64 ± 6.47	<0.0001
Year of diagnosis			<0.0001
≤ 2000	259294	10029	
2001-2005	145788	2937	
2006-2015	143616	1634	
Sex			<0.0001
Female	280520	6186	
Male	268178	8414	
Race			<0.0001
White	456866	12561	
Black	52277	1087	
Other/ unknow	39555	952	
Number of primary tumors			<0.0001
One	387407	10700	
Multiple	161291	3900	
Surgery			<0.0001
No	32715	301	
Yes	515983	14299	
Radiotherapy			<0.0001
No/unknown	64582	770	
Yes	484116	13830	
Chemotherapy			<0.0001
No/unknown	162366	1609	
Yes	386332	12991	

Grading[a]			<0.0001
I	67579	2270	
II	365787	9755	
III	107471	2404	
IV	7861	171	
Stage[a]			<0.0001
In situ	348	8	
Localized	217044	7737	
Regional	228432	6237	
Distant	102874	618	
Tumor site			<0.0001
Cecum, appendix and ascending colon	160718	4717	
Transverse colon and hepatic or splenic flexure of colon	70116	2133	
Descending colon, sigmoid colon and rectosigmoid junction	193771	5071	
Rectum	97931	1993	
Overlapping lesion or tumors involving multiple locations	26162	686	

CVSM, cerebrovascular-specific mortality; CRC, colorectal cancer; SD, standard deviation; [a]analyzed by Wilcoxon's rank sum test.

Supplementary Table S2. Year of diagnosis-specific and overall standardized mortality ratios for the years 1999-2015 among colorectal cancer relative to the USA standard population

Group	cSMR cerebrovascular-specific	*P*-value	cSMR overall	*P*-value
Year of diagnosis				
≤ 2000	62.29 (61.06-63.54)	< 0.0001	258.66 (257.60-259.73)	< 0.0001
2001-2005	36.85 (35.50-38.22)	< 0.0001	216.76 (215.39-218.14)	< 0.0001
2006-2015	26.32 (25.03-27.64)	< 0.0001	197.39 (195.94-198.85)	< 0.0001

cSMR, conditional standardized mortality ratio.

Supplementary Table S3. Correction between individual prognostic factors of CVSM among CRC patients

		Year	Sex	Race	Number of primary tumors	Surgery	Chemotherapy	Radiotherapy	Grading	Stage	Age	Tumor site
Year	r	1	-0.008**	0.061**	-0.019**	0.055**	-0.146**	-0.036**	0.028**	-0.032**	-0.054**	-0.038**
	P value		0	0	0	0	0	0	0	0	0	0
Sex	r	-0.008**	1	0.003*	-0.033**	-0.019**	0.042**	0.061**	0.035**	0.004**	0.090**	-0.093**
	P value	0		0.029	0	0	0	0	0	0.001	0	0
Race	r	0.061**	0.003**	1	-0.042**	0.026**	-0.042**	-0.007**	-0.019**	0.024**	-0.108**	0.010**
	P value	0	0.029		0	0	0	0	0	0	0	0
Number of primary tumors	r	-0.019**	-0.033**	-0.042**	1	-0.047**	0.081**	0.052**	-0.027**	-0.122**	0.111**	0.078**
	P value	0	0	0		0	0	0	0	0	0	0
Surgery	r	0.055**	-0.019**	0.026**	-0.047**	1	-0.067**	-0.110**	0.037**	0.187**	0.009**	0.102**
	P value	0	0	0	0		0	0	0	0	0	0
Chemotherapy	r	-0.146**	0.042**	-0.042**	0.081**	-0.067**	1	0.383**	-0.123**	-0.341**	0.292**	-0.095**
	P value	0	0	0	0	0		0	0	0	0	0
Radiotherapy	r	-0.036**	0.061**	-0.007**	0.052**	-0.110**	0.383**	1	-0.341**	-0.073**	0.163**	-0.340**
	P value	0	0	0	0	0	0		0	0	0	0

Grading	r	0.028**	0.035**	-0.019**	-0.027**	0.037**	-0.123**	-0.034**	1	0.232**	0.013**	-0.079**
	P value	0	0	0	0	0	0	0		0	0	0
Stage	r	-0.032**	0.004**	0.024**	-0.122**	0.187**	-0.341**	-0.073**	0.232**	1	-0.066**	-0.065**
	P value	0	0	0	0	0	0	0	0		0	0
Age	r	-0.054**	0.090**	-0.108**	0.111**	0.009**	0.292**	0.163**	0.013**	-0.066**	1	-0.141**
	P value	0	0	0	0	0	0	0	0	0		0
Tumor site	r	-0.038**	-0.093**	0.010**	0.078**	0.102**	-0.095**	-0.340**	-0.079**	-0.065**	-0.141**	1
	P value	0	0	0	0	0	0	0	0	0	0	

*indicates $P < 0.05$, **indicates $P < 0.001$. r, correlation coefficient.
